# Pro-inflammatory genetic profile and familiarity of acute myocardial infarction

**DOI:** 10.1186/1742-4933-9-14

**Published:** 2012-06-24

**Authors:** Manuela Ianni, Sergio Callegari, Antonio Rizzo, Paolo Pastori, Paolo Moruzzi, Domenico Corradi, Elisa Porcellini, Gianluca Campo, Roberto Ferrari, Marco M Ferrario, Stefania Bitonte, Ilaria Carbone, Federico Licastro

**Affiliations:** 1 Department of Experimental Pathology, School of Medicine, University of Bologna, Via S. Giacomo 14, 40126, Bologna, Italy; 2 Fidenza Hospital, Division of Cardiology, AUSL, Parma, Italy; 3 School of Cardiology, University of Parma, Parma, Italy; 4 Department of Pathology and Laboratory Medicine, Section of Pathology, University of Parma, Parma, Italy; 5 Department of Cardiology, University of Ferrara, Salvatore Maugeri Foundation, IRCCS, Lumezzane and Department of Morphology and Embryology and LTTA Centre, University of Ferrara, Ferrara, Italy; 6 Department of Experimental Medicine, University of Insubria, Varese, Italy

**Keywords:** Acute myocardial infarction, Family history, Genetic association, Inflammation

## Abstract

**Background:**

Acute myocardial infarction (AMI) is a multifactorial disease with a complex pathogenesis where lifestyle, individual genetic background and environmental risk factors are involved. Altered inflammatory responses are implicated in the pathogenesis of atherosclerosis and a premature AMI of parents is associated with an increased risk of the disease in their offspring (Offs). However, the genetic background of familiarity for AMI is still largely unknown. To understand which genes may predispose to increased risk of cardiovascular disease gene polymorphism of immune regulatory genes, and clinical events from the Offs of parents with an early AMI were investigated. Genetics data from Offs were compared with those obtained from healthy subjects and an independent cohort of patients with clinical sporadic AMI. Rates of clinical events during a 24 years follow up from Offs and from an independent Italian population survey were also evaluated.

**Results:**

This study showed that a genetic signature consisting of the concomitant presence of the CC genotype of VEGF, the A allele of IL-10 and the A allele of IFN-γ was indeed present in the Offs population. In fact, the above genetic markers were more frequent in unaffected Offs (46.4%) and patients with sporadic AMI (31.8%) than in the CTR (17.3%) and the differences were highly statistically significant (Offs vs CTR: p = 0.0001, OR = 4.129; AMI vs CTR: p = 0.0001, OR = 2.224). During the 24-year follow-up, Offs with a positive familiarity in spite of a relatively young age showed an increased prevalence of diabetes, ischemic heart disease and stroke. These findings reinforce the notion that subjects with a familial history of AMI are at risk of an accelerated aging of cardiovascular system resulting in cardiovascular events.

**Conclusion:**

Our data suggest that selected genes with immune regulatory functions are part of the complex genetic background contributing to familiarity for cardiovascular diseases. This inflammatory genetic profile, along with classical cardiovascular risk factors, may be used for better defining individual risk of AMI in unaffected subjects.

## Background

Knowledge of the etiology and pathogenetic mechanisms of coronary heart disease (CHD) is still limited and incomplete, and more than 50% of CHD patients do not have the classical risk factors of hypercholesterolemia, hypertension, smoking, diabetes, obesity or a sedentary lifestyle [1]. CHD is the most frequent cause of morbidity and mortality worldwide [2], and acute myocardial infarction (AMI) is the main clinical complication of CHD.

AMI is a multifactorial disease with a complex pathogenesis in which lifestyles, individual genetic backgrounds and environmental risk factors contribute to the pathogenetic mechanisms and clinical manifestations: for example, abnormal blood lipid and lipoprotein levels [3,4] along with altered immune factors promote atherogenesis and lead to AMI [5,6].

It has been suggested that biomarkers of inflammation, such as increased blood homocysteine [7], C-reactive protein [6], and cytokine levels [8-12], may be new risk factors for CHD. However, phenotype biomarkers can vary widely in the same person as a function of time and may be different among subjects as result of gender, concomitant diseases, metabolic disorders, dietary intake and other environmental variables.

Inherited gene variants are less influenced by environment factors, and may be a better marker of individual AMI susceptibility, particularly in cases of familial AMI. A family history of cardiovascular diseases (CVD) and AMI is frequently encountered in clinical practice, and a premature heart attack in parents is associated with a high risk of the disease in their offspring (Offs) [13]. This initial observation has been confirmed and extended by other studies showing that parental CVD is a risk factor for CVD in middle-aged Offs [14]. The relevance of familial factors to CVD is further supported by the finding that siblings with CVD are at increased risk of future cardiovascular events (CVE) regardless of the presence of established risk factors [15]. It has also been shown that parental longevity relates to carotid atherosclerosis and aortic arterial stiffness in adult Offs [16]. Data from the longitudinal Framingham Heart Study confirm that subjects with long-lived parents have a better cardiovascular risk profile in middle age than those whose parents died younger [17] and a low cardiovascular risk profile appears to contribute to the longevity of centenarians [18]. Furthermore, the Offs of centenarians have a better cardiovascular risk profile than those of parents not enjoying a long life [19]. It is interesting to note that structural vascular changes can be observed in Offs with a parental history of AMI at a young age, regardless of the presence of a number of the classical cardiovascular risk factors [20]. Once again, the Framingham Heart Study has shown that parental stroke before the age of 65 years is associated with a 3-fold increase in the risk of stroke in their Offs, and that parental history can be used as clinical risk marker of an individual’s propensity to stroke [21].

All of these findings suggest that genetic studies of Offs may be useful to identifying transmissible genetic traits in CVD.

We hypothesized that studying unaffected subjects with positive familiarity for AMI may improve our understanding of the type of genetic background involved in the development of atherosclerosis and its complications. Therefore, we investigated genetic variations represented by single nucleotide polymorphisms (SNPs) in the promoter region of a number of genes regulating metabolic and immune functions that have previously been found to be associated with an increased risk of AMI in case/control studies [22].

Genetic data obtained from Offs followed up for 24 years, unrelated patients with clinical AMI, and elderly controls (CTR) without a history of CVD revealed a common multi-gene profile in the AMI cases and unaffected Offs with a positive parental history of AMI, which consisted of SNPs of the VEGF, IL-10, IFN-γ genes. Offs indeed showed an increased prevalence of CVD. The pro-inflammatory genetic profile described here may be useful for predicting individual risk in unaffected subjects.

## Results

### Genotype and allele frequency

Table 1shows the SNP numbers, gene positions and mutated alleles of the investigated VEGF, ACT, HMG-CR, IL-1β, IL-10 and IFN-γ genes, together with the number, mean age and gender of the subjects in the different groups.

**Table 1:** SNPs list and the number, age and gender distribution of the CTR, Offs and AMI

**Gene polymorphism:**			
VEGF (rs 699947)		SNP at −2578, allele mutation = A	
IL-10 (rs 1800896)		SNP at −1082, allele mutation = A	
IFN-γ (rs 2430561)		SNP at +874, allele mutation = A	
ACT (rs 1884082)		SNP at −51, allele mutation = T	
HMG-CR (rs 3761740)		SNP at −911, allele mutation = A	
IL-1β (rs 16944)		SNP at −511, allele mutation = T	
	**N**	**Mean age**	**Gender**
CTR	321	72.0 ± 5.1	158 M/163 F
Offs	154	55.8 ± 6.7	80 M/74 F
AMI	267	67.7 ± 12.2	195 M/72 F

The genotype distribution and allele frequency of the VEGF gene are shown in Table 2. The CC genotype was more frequent in the Offs than the CTR (63% *vs* 40.9%, p = 0.0001), and also more frequent in the AMI group (65% *vs* 40.9%, p = 0.0001; OR = 2.689). The percentage of VEGF C carriers was significantly higher in the AMI group than in the CTR (95.3% *vs* 84.5%, p = 0.0001; OR = 3.735), whereas the percentage of A carriers was significantly lower in the Offs (37.7%, p = 0.0001) and the AMI group (35%, p = 0.0001; OR = 0.373) than the CTR (59.1%). The AA genotype was more frequent in the CTR than in the AMI group (15.5% *vs* 4.7%, p = 0.0001; OR = 0.269).

**Table 2:** Genotype distribution and allele frequency of VEGF SNP (rs 699947) from Offs, CTR and AMI

**VEGF**	**CC (n) %**	**CA (n) %**	**AA (n) %**	**C carriers (n) %**	**A carriers** (n) %
**Offs (n = 154)**	(97) 63	(42) 27.3	(15) 9.7	(139) 90.3	(58) 37.7
**CTR (n = 291)**	(119) 40.9	(127) 43.6	(45) 15.5	(246) 84.5	(172) 59.1
**AMI (n = 257)**	(167) 65	(78) 30.4	(12) 4.7	(245) 95.3	(90) 35
**Offs*vs*CTR**	*χ*^2^ = 19.680, p = 0.0001; CC carriers *vs* non-CC carriers: *χ*^2^ = 19.745, p = 0.0001; A carriers *χ*^2^ = 18.545, p = 0.0001.				
**AMI*vs*CTR**	*χ*^2^ = 36.906, p = 0.0001; CC carriers *vs* non-CC carriers: *χ*^2^ = 31.796, p = 0.0001; OR = 2.689 (CI: 1.900-3.806); AA carriers *vs* non-AA carriers: *χ*^2^ = 16.943, p = 0.0001; OR = 0.269 (CI: 0.139-0.521); C carriers *χ*^2^ = 17.063, p = 0.0001; OR = 3.735 (CI: 1.929-7.232); A carriers *χ*^2^ = 31.733, p = 0.0001; OR = 0.373 (CI: 0.264-0.527).				
**Offs*vs*AMI**	*χ*^2^ = 4.141, p = 0.126.				

Table 3 shows the IL-10 genotype distribution and allele frequency in the three groups. The GG genotype was more frequent in the CTR than in the Offs (30.5% *vs* 13%, p = 0.0001) or the AMI group (21.1%, p = 0.016; OR = 0.609). The A allele was significantly less frequent in the CTR than in the Offs (69% *vs* 87.7%, p = 0.0001) or the AMI group (78.9%, p = 0.012; OR = 1.674). There were also some differences between the Offs and the AMI group: the GG genotype was more frequent in the latter (21.1% *vs* 13%, p = 0.037; OR = 1.795); there was a higher percentage of A carriers among the Offs (87.7% *vs* 78.9%, p = 0.02), in whom the frequency of the AA genotype was also higher (35.1% *vs* AMI =25.7%, p = 0.043).

**Table 3:** IL-10 SNP (rs 1800896) genotype distribution and allele frequency from Offs, CTR and AMI

**IL-10**	**GG (n) %**	**GA (n) %**	**AA (n) %**	**G carriers (n) %**	**A carriers (n) %**
**Offs (n = 154)**	(20) 13	(80) 51.9	(54) 35.1	(101) 65.6	(135) 87.7
**CTR (n = 239)**	(73) 30.5	(88) 36.8	(78) 32.6	(161) 67.4	(165) 69
**AMI (n = 265)**	(56) 21.1	(141) 53.2	(68) 25.7	(197) 74.3	(209) 78.9
**Offs*vs*CTR**	*χ*^2^ = 17.378, p = 0.0001; GG carriers *vs* non-GG carriers: *χ*^2^ = 15.981, p = 0.0001; A carriers *χ*^2^ = 17.984. p = 0.0001				
**AMI*vs*CTR**	*χ*^2^ = 13.887, p = 0.001; GG carriers *vs* non-GG carriers: *χ*^2^ = 5.845, p = 0.016; OR = 0.609 (CI: 0.407-0.912); A carriers *χ*^2^ = 6.344, p = 0.012; OR = 1.674 (CI: 1.179-2.504)				
**Offs*vs*AMI**	*χ*^2^ = 6.550, p = 0.038; GG carriers *vs* non-GG carriers: *χ*^2^ = 4.352, p = 0.037; OR = 1.795 (CI: 1.031-3.126); AA carriers *vs* non-AA carriers: *χ*^2^ = 4.077, p = 0.043; OR = 0.642 (CI: 0.418-0.989); A carriers *χ*^2^ = 5.126, p = 0.024; OR = 0.525 (CI: 0.299-0.923).				

Table 4 shows IFN-γ genotype and allele distribution. The TT genotype was more frequent in the CTR than in the Offs (30.6% *vs* 15.7%, p = 0.001), whereas the percentage of A carriers was higher among the Offs than the CTR (84.3% *vs* 69.4%, p = 0.001). No difference in the distribution of the IFN-γ polymorphism between the AMI group and the CTR was detected, but the frequency of the TT genotype was slightly higher in the AMI group than in Offs (24.2% *vs* 15.7%, p = 0.036; OR = 1.761) and the A allele was less frequent in the AMI group than Offs (75.8% *vs* 84.3%, p = 0.044; OR = 0.584).

**Table 4:** SNP IFN-γ (rs 2430561) genotype distribution and allele frequency from Offs, CTR and AMI.

**IFN-γ**	**TT (n) %**	**TA (n) %**	**AA (n) %**	**T carr (n) %**	**A carr (n) %**
**Offs (n = 153)**	(24) 15.7	(88) 57.5	(41) 26.8	(112) 73.2	(129) 84.3
**CTR (n = 268)**	(82) 30.6	(111) 41.4	(75) 28	(193) 72.0	(186) 69.4
**AMI (n = 240)**	(58) 24.2	(96) 40	(86) 35.8	(154) 64.2	(182) 75.8
**Offs vs CTR**	*χ*^2^ = 13.990, p = 0.001; TT carriers *vs* non-TT carriers: *χ*^2^ = 11.070, p = 0.001; A carriers *χ*^2^ = 11.495, p = 0.001				
**AMI*vs*CTR**	*χ*^2^ = 4.423, p = 0.110				
**Offs*vs*AMI**	*χ*^2^ = 11.704, p = 0.003; TT carriers *vs* non-TT carriers: *χ*^2^ = 4.380, p = 0.036; OR = 1.761 (CI: 1.032-3.002); A carriers *χ*^2^ = 4.070, p = 0.044; OR = 0.584 (CI: 0.345-0.988).				

The SNPs in the promoter region of the ACT, HMG-CR and IL-1β genes were also investigated, but no statistically significant difference in allele and genotype frequencies between the groups was found (data not shown).

### Association between the triple genotype and cardiovascular risk

The concomitant presence of the CC genotype of VEGF, the A allele of IL-10 and the A allele of IFN-γ was also determined and resulted to be associated with an increased risk of AMI, as shown in Table 5. This “triple genotype” was more frequent in the Offs (46.4%) and the AMI (31.8%) than in CTR (17.3%), and the differences were highly statistically significant (Offs *vs* CTR: p = 0.0001, OR = 4.129; AMI *vs* CTR: p = 0.0001, OR = 2.224).

**Table 5:** Concomitant presence of the triple genotype in Offs, CTR and AMI

**Triple genotype**	**Carriers (n) %**	**Non-carriers (n) %**
**Offs (n = 153)**	(71) 46.4	(82) 53.6
**CTR (n = 301)**	(52) 17.3	(248) 82.7
**AMI (n = 239)**	(76) 31.8	(163) 68.2
**Offs*vs*CTR**	*χ*^2^ = 43.295, p = 0.0001	
**AMI*vs*CTR**	*χ*^2^ = 15.372, p = 0.0001; OR = 2.224 (CI: 1.484-3.332)	

### Body mass index (BMI) and blood lipid profile

Data regarding BMI and serum lipid profile from Offs, CTR and IMA have been reported in Table 6. BMI values of Offs and IMA were slightly increased (27 ± 4) as compared with ideal age matched reference value. Moreover, BMI from our CTR group was higher than Offs and IMA, however this difference may be mainly ascribed to the older age of CTR.

**Table 6:** BMI values and blood lipid profiles from Offs, CTR and AMI.

		**N°**	**Mean**	**St. Deviation**	**Post hoc statistics**
**BMI**	Offs	154	27	4	Offs vs CTR*
	CTR	320	29	4	Offs vs IMA**
	IMA	265	27	4	CTR vs IMA*
**Total cholesterol**	Offs	148	216	43	Offs vs CTR*
	CTR	265	241	39	Offs vs IMA*
	IMA	209	199	39	CTR vs IMA*
**HDL**	Offs	148	60	17	Offs vs CTR**
	CTR	265	60	15	Offs vs IMA*
	IMA	209	47	16	CTR vs IMA*
**LDL**	Offs	137	132	36	Offs vs CTR*
	CTR	241	157	34	Offs vs IMA**
	IMA	189	127	38	CTR vs IMA*
**Triglycerides**	Offs	148	111	64	Offs vs CTR**
	CTR	265	122	70	Offs vs IMA*
	IMA	209	140	117	CTR vs IMA*
**VLDL**	Offs	148	22	13	Offs vs CTR**
	CTR	265	24	14	Offs vs IMA*
	IMA	209	28	23	CTR vs IMA*

*p = 0.0001.

**p ≥ 0.05; not statistically significant.

Lipid profile from the Offs population was substantially in the normal range for their age cohort. Once again CTR population showed slightly increased blood levels of total cholesterol, LDL and triglycerides as expected according their age range.

As shown in Table 7, no statistical difference for BMI, total cholesterol, HDL, LDL, triglycerides and VDL values between Offs carriers and non carriers for the triple genotype was detected.

**Table 7:** BMI and blood lipid parameters in Offs carriers or non carriers of the triple genotype.

	**Triple genotype Offs**	**N°**	**Mean**	**St. Deviation**	**statistics**
**BMI**	Carriers	71	26	4	not significant
	Not carriers	82	27	4	
**Total cholesterol**	Carriers	66	215	35	not significant
	Not carriers	81	215	47	
**HDL**	Carriers	66	60	14	not significant
	Not carriers	81	60	18	
**LDL**	Carriers	64	134	31	not significant
	Not carriers	72	130	38	
**Triglycerides**	Carriers	66	100	53	not significant
	Not carriers	81	120	74	
**VLDL**	Carriers	66	20	11	not significant
	Not carriers	81	24	15	

### Prevalence of cardiovascular events (CVE) after 24 years of follow up

The prevalence rates of a history of ischemic heart disease (AMI or angina pectoris), stroke, ischemic heart disease plus stroke, hypertension, diabetes and smoking in 154 Offs at the beginning (age 23–35 years; Table 8, panel A) and at the end of the follow up period (age 50–60 years; Table 8, panel B) were compared with those of gender and age matched subjects from the MONICA-Brianza population. No difference in the event rates was present between the two populations at the beginning of the follow up period. On the contrary, at the end of the follow up the prevalence of ischemic heart disease among the male Offs was three times higher, that of stroke was eight times higher, that of stroke and ischemic heart disease was three times higher, and that of diabetes was twice as high. However, there was no increased prevalence of CVE among female Offs during the same period.

**Table 8:** Cardiovascular events in Controls and Offs during a 24 years follow up.

**panel A**										
	**Male**					**Female**				
	MONICA-Brianza		Offs		P	MONICA-Brianza		Offs		P
	Age 25-35		(1984)		†	Age 25-35		(1984)		†
	N	%	N	%		N	%	N	%	
Subjects	727	-	84	-	-	768	-	70	-	-
Ischemic heart disease*	3	0.4	1	1.2	‡	2	0.3	0	0	‡
Stroke	1	0.1	0	0	‡	0	0	0	0	‡
Heart disease/stroke	4	0.6	1	1.2	‡	2	0.3	0	0	‡
Hypertension	72	9.9	4	4.8	‡	63	8.2	2	2.9	‡
Diabetes mellitus	2	0.3	0	0	‡	2	0.3	1	1.4	‡
Smoking	292	40.2	29	34.5	‡	240	31.3	27	38.6	‡
**panel B**										
	MONICA-Brianza		Offs		P	MONICA-Brianza		Offs		P
	Age 50-60		(2008)		†	Age 50-60		(2008)		†
	N	%	N	%		N	%	N	%	
Subjects	970	-	84	-	-	967	-	70	-	-
Ischemic heart disease*	38	3.9	9	10.7	0.01	16	1.7	1	1.4	‡
Stroke	6	0.6	4	4.8	0.01	3	0.3	0	0	‡
Heart disease/stroke	44	4.5	13	15.5	0.0001	19	2	1	1.4	‡
Hypertension	268	27.6	29	34.5	‡	350	36.2	20	28.6	‡
Diabetes mellitus	39	4	8	9.5	0.05	30	3.1	5	7.1	‡
Smoking	354	36.5	17	20.2	0.003	145	15	13	18.6	‡

## Discussion

The multiple pathogenetic pathways leading to AMI include genetic heterogeneity or multiple genetic traits associated with the disease. Recent genome-wide association (GWA) studies have contributed substantially to the discovery of new SNPs associated with CHD and AMI [23], but their clinical relevance is still unclear because a single gene variant can make a limited contribution to the total genetic load of AMI, and both common and rare gene polymorphisms may differentially affect susceptibility to the disease. These factors may also partially explain the contradictory results of genetic association studies using the candidate gene approach in AMI case/control studies [24-26].

It is important to know that subjects with an affected parent have a two-fold greater risk of CHD than those without a family history [13-15]. Genetic studies of the children of parents with CVD have shown that genetic variations in the promoter region of the APOA1 gene are associated with differences in serum ApoA1 and HDL levels in healthy subjects, and that this effect is influenced by gender and a family history of AMI [27]. A parental history of hypertension is a risk factor for high blood pressure among Offs, whose blood pressure is partially affected by variations (deletions/insertions) in the angiotensin converting enzyme [28]. Hyper-homocysteinemia is frequent in homozygotes for the C677T polymorphism of the MTHFR gene and associated with an increased risk of CHD in children with a positive family history [29]. It has also been found that the same SNP is associated with congenital atrial septal defects, and that these heart alterations are more frequent in the children from mothers carrying the MTHFR TT genotype [30].

It is known that a family history and a still largely undefined genetic background greatly influence the early clinical manifestation of AMI and CVD. Therefore, we investigated SNPs in genes with a regulatory effect on inflammatory responses as possible genetic markers of an increased risk of CVD in children of parents with a positive history of AMI. It is interesting that 45% of the Offs in our investigation had a father who suffered an AMI before he reached the age of 56 years.

We chose elderly CTR from a longitudinal population study because they did not have a history of CVD, and did not experience an AMI or have any other CVD before and during the five years of follow-up. A second control group of comparable age from the WHO-MONICA-Brianza study to compare the CVE prevalence during the 24 years follow-up of our Offs was also used. The control disease consisted of patients with clinical sporadic AMI.

SNPs in the VEGF, IL-10 and IFN-γ genes were differently distributed in Offs and CTR, and it is interesting to note that the genetic make-up of the Offs overlapped that of the unrelated population of patients with a clinical diagnosis of sporadic AMI (control disease).

It has been suggested that the CC genotype of the VEGF gene, together with two other SNPs in the promoter region of this gene, increases VEGF gene expression in human myoblasts [31] and the production of the cognate protein in human peripheral blood lymphocytes activated by lipopolisaccaride [32]. Subjects with the VEGF CC genotype may produce increased levels of VEGF protein which, by deregulating angiogenesis, may lead to an increased risk of CVE.

The published data regarding the functional relevance of IL-10 SNPs are conflicting. An initial study found that the IL-10 A allele is associated with a two-fold increase in transcriptional activity in B cell lines [33], but it has been subsequently reported that the A allele is associated with a reduction in the IL-10 secretion of activated peripheral blood lymphocytes [34-36]. These data support the hypothesis that the suppression of inflammation may be impaired in carriers of the −1082 A allele in the IL-10 gene. These findings suggest that impaired regulation of inflammatory responses in Offs with one or two copies of the IL-10 A allele may increase the risk of CVE.

The +874 A allele of the IFN-γ gene decreases the production of the cognate protein and resistance to tuberculosis infection [37]. Our findings showing an increased frequency of the +874 A allele in Offs are in accordance with another study reporting that patients with the +874 AA genotype and idiopathic dilated cardiomyopathy showed a worse prognosis and an adverse outcome [38]. Furthermore, patients with dilated cardiomyopathy showed an impaired activation of CD4T cells by IFN-γ ascribed to a decreased production of this cytokine [39]. These findings suggest that a decreased release of IFN-γ may negatively affect the coordination of immune responses in vessel walls, accelerate atherogenesis, and increase the risk of CVE in Offs with the IFN-γ A allele.

The concomitant presence of the CC genotype of the VEGF gene, the A allele of the IL-10 gene, and the A allele of the IFN-γ gene was more frequent in our AMI group, and significantly increased the risk of the disease. It is interesting to note that this triple genotype profile was found in 46% of Offs and led to a presumptive high risk of CVE (OR = 4.129). This observation supports the notion that Offs with a positive parental history are at high risk of CVE and the pro-inflammatory genetic signature may be part of the complex genetic background influencing AMI risk.

The increased risk of CVE predicted by genetic signature was partially confirmed by the 24-year follow-up, which clearly revealed a significant increase in CVE in Offs. Furthermore, gender and age are also strong risk factors, as CVE increased prevalence was only observed in older male Offs (Table 8). The type of relationship between the presence of the triple genotype and CVE manifestation could not be assessed, since the limited number of Offs positive for CVE. Further follow up of these subjects may clarify this topic.

BMI from Offs was slightly increased when compared with the ideal age value and CTR selected for this investigation showed an increased BMI as expected since their advanced age. Cholesterol blood profile data from Offs group were in the normal range for their age cohort. Among Offs only 33 subjects used for limited time periods statins and the number of statins users was further reduced when it was stratified between triple genotype carriers and non carriers. Therefore, statins use showed very limited influence upon the lipid profile data from Offs population. Furthermore, since the presence of the triple genotype/allele signature did not affect BMI or the blood levels of total cholesterol, HDL, LDL or triglycerides, we suggest that it might affect the incidence of CVE by mechanisms that are partially independent of those affected by the classic risk factors of CVD.

## Conclusions

The concomitant presence of the CC genotype of VEGF, the A allele of IL-10 and the A allele of IFN-γ resulted to be associated with an increased risk of AMI and was more frequent among Offs with a positive parental history of AMI. Therefore, genes with an immune regulatory function appear to be associated in the pathogenesis of AMI. Inflammatory genes, gender and age influence an accelerated aging of cardiovascular system and selected genes with immune regulatory functions are part of the complex genetic background contributing to familiarity for cardiovascular diseases.

## Methods

### Subjects and patients

The study involved 154 Offs from Northern Italy, each of whom had one parent who had experienced an AMI before the age of 65 years. An evaluation was made of the classic AMI risk factors (metabolic parameters, smoking, diabetes, obesity, a sedentary lifestyle) together with ECG records, anthropometric indices, arterial pressure and medical history. The Offs were re-screened after a follow-up of 24 years in order to assess any changes in behavioral and biological risk factors and collect blood samples for laboratory and genetic evaluation. The CVD risk factors and drug treatments recorded at baseline and 24 years later were compared with those of the age-matched population of the WHO-MONICA Project, which were collected in Brianza, Northern Italy, during independent surveys carried out in 1984, 1991 and 2004 [40]. A further group of 269 consecutive patients with a clinical diagnosis of AMI based on electrocardiographic changes and standard laboratory findings, and confirmed by echocardiography and coronary angiography [22], who were admitted to the Cardiology Unit of Ferrara University Hospital during 2006–2007, was investigated. The healthy CTR were 315 subjects without a family history of CVD participating in the “Conselice study of brain aging” conducted in Northern Italy in 1999–2005 [41], none of whom showed any signs of CVD or inflammatory diseases before or during the study.

The plasma cholesterol and lipid profiles of the Offs, AMI patients and CTR were determined on the basis of standard laboratory procedures.

The research protocol was approved by our Institutional Review Boards, and all of the participants gave their written informed consent.

### DNA extraction

Genomic DNA was extracted from peripheral blood leukocytes as described elsewhere [42].

### SNP detection

The presence of SNPs in the promoter regions of the VEGF (−2578 C/A), ACT (−51 G/T) and HMG-CR genes (−911 C/A) was assessed by means of polymerase chain reaction (PCR)-based methods as previously described [43-45]. SNPs in the IL-1β (−511 C/T), IL-10 (−1082 G/A) and IFN-γ genes (+874 T/A) were detected by real-time PCR. The SNP-specific primers and probes were designed using the TaqMan genotyping assay (ABI, Foster City, CA) in a 25 μl total volume of BIORAD CFX 96 in accordance with the manufacturer’s instructions [22].

### Statistical analysis

The different genotypes were statistically analyzed using contingency tables and the chi-square (*χ*2) test, and the odds ratios (OR) and their statistical significance were also calculated. The mean values of the various quantitative variables were compared by means of one-way analysis of variance (ANOVA) followed by appropriate post-hoc comparisons and Bonferroni’s correction. Statistical tests were two-sided, and significance was set at p < 0.05.

## Abbreviations

CHD, Coronary heart disease; AMI, Acute myocardial infarction; CVD, Cardiovascular diseases; CVE, Cardiovascular events; Offs, Offspring; CTR, Controls.

## Competing interests

The authors declare that they have no competing interests.

## Authors’ contributions

MI, EP, SB, IC performed laboratory analysis and genotyping; MI also performed statistical analysis of data and contributed to draft the article; FL contributed to design the clinical, epidemiological and genetic study and wrote the discussion of the article; SC, AR, PP, PM, DC enrolled offspring with at least one parent affected by acute myocardial infarction hospitalized at the Cardiology Unit of Fidenza Hospital, Parma, Italy during the period 1974–1983, collected blood samples and data regarding the clinical history of Offs and contributed to design the clinical and epidemiological investigation. GC and RF enrolled patients with acute myocardial infarction admitted to the Cardiology Unit of Ferrara University Hospital during the period 2006–2007, collected blood samples and data regarding clinical history of patients with clinical diagnosis of AMI. MMF collected epidemiological data from the MONICA (MONItoring of CArdiovascular diseases) Project conducted in Brianza and contributed to the paper discussion. All authors read and approved the final manuscript.

## Funding

This study was supported by the Italian Ministry of Universities and Research, the National Institute for Cardiovascular Disease, the Carisbo Foundation, and Chiesi Farmaceutici, Italy.
